# Achieving Lumbar Epidural Block Competency in Inexperienced Trainees after a Structured Epidural Teaching Model: A Randomized, Single Blind, Prospective Comparison of CUSUM Learning Curves

**DOI:** 10.1155/2022/1738783

**Published:** 2022-09-02

**Authors:** Marco Scorzoni, Gian Luigi Gonnella, Emanuele Capogna, Matteo Velardo, Pietro Paolo Giuri, Mariano Ciancia, Giorgio Capogna, Gaetano Draisci

**Affiliations:** ^1^Department of Anesthesiology, Catholic University Medical School-Fondazione Policlinico Gemelli IRCCS, Roma, Italy; ^2^European School of Obstetric Anesthesia, Roma, Italy

## Abstract

**Aim:**

The aim of this randomized, prospective study was to investigate whether the use of the structured epidural teaching model (SETM) may affect the learning curve for lumbar epidural block in novice trainees when compared with a standard teaching module.

**Introduction:**

There is a paucity of literature regarding the efficacy of teaching epidural blocks and comparisons between the different educational approaches.

**Method:**

Forty-four PGY3 anesthesia trainees were randomized to receive (study group) or to not receive (control group) the SDM (structured didactic model) before the beginning of their 6 months clinical practice rotation in labor and delivery suites. A CUSUM learning curve was built for every trainee. The scores were assigned by the staff instructor, who was unaware of the group to which the trainee belonged.

**Results:**

The number of subjects who achieved an improvement in performance was 8 trainees from the control group and 14 from the study group. The probability of achieving an improvement was higher (*p* < 05) in the study group than in the control group, with an aOR of 3.25 (CI: 1.01; 12.1). The proportion of subjects in the study group who completed the epidural without help was 1.21 (1.05–1.41) times the proportion of subjects who completed the epidural without help in the control group. The probability of completing the epidural block without any assistance was 21% higher in the study group than in the control group (*p* < 05).

**Conclusion:**

We have demonstrated that the use of the structured epidural teaching model (SETM) may improve the learning curve (CUSUM) for lumbar epidural block in novice, entirely inexperienced, anesthesia trainees.

## 1. Introduction

In the past, the typical teaching of epidural catheter placement consisted of a combination of didactic education and hands-on experience, where trainee and master approached the task together in the clinical setting, with live patients as the learning model. Nowadays, in some hospitals, novice anesthesiologists still undergo training directly on patients, while in others they use simulation and simulators before going to the patient.

Low and high-fidelity simulators [1], videotaping [2, 3], e-learning tools [4, 5], and computer-enhanced visual learning [6] have been proposed and may represent promising and useful teaching tools.

Checklists have been found to have excellent reliability in the assessment of epidural anesthesia even if published checklists greatly differ between them, having so many different items on their lists [7–9]. In addition, they reflect local practice and may be impractical in different institutions worldwide.

Furthermore, there is a paucity of literature regarding the efficacy of such a method and the comparison between the different educational approaches.

As in many hospitals worldwide, at the Catholic University Medical School-Fondazione Policlinico Gemelli IRCCS in Rome, anesthesia trainees, after having received a standard lecture on epidural anatomy and technique, undergo an epidural technique training directly on patients in the labor and delivery setting, under the supervision of an instructor.

A structured epidural teaching model (SETM) practiced and taught since the 1990s has now been fully updated and described in detail [10]. It includes three standardized video lessons, the construction of a 3D epidural module by trainees and practical training by using an epidural simulator with and without the CompuFlo™ Epidural Trainer instrument [11].

The aim of this randomized, prospective study was to investigate whether the use of the structured epidural teaching model (SETM) may affect the lumbar epidural block learning curve in novice, entirely inexperienced, anesthesia trainees when compared with a standard teaching module by using cumulative sum (CUSUM) analysis [12, 13] and linear regression models.

## 2. Methods

The study protocol was registered at Clinical. Trial. Gov (ID *n*. NCT04749186) and was approved by the Ethics Committee of Fondazione Policlinico Gemelli IRCCS, Rome (approval *n* 0017199/21).

Forty-four PGY3 anesthesia trainees from the Catholic University Medical School-Fondazione Policlinico Gemelli IRCCS accepted to be enrolled in this randomized, prospective, single blinded, observational study. Each participant gave written informed consent, and privacy, confidentiality and anonymity were fully guaranteed.

Only trainees who had never previously performed an epidural block and were about to begin their obstetrics rotation were enrolled in this study.

After having had the usual institutional lecture on epidural anatomy and technique, followed by a nonstandardized four hour practice on an epidural simulator, the subjects were randomized to receive (study group; *n* = 22) or not to receive (control group; *n* = 22) the SDM (Structured Didactic Model) before the beginning of their 6 months clinical practice rotation in the labor and delivery suites.

### 2.1. Structured Epidural Teaching Model (SETM)

The study group received the structured epidural teaching model (SETM) [10]. This model includes three modules.

#### 2.1.1. Module

The first module aimed to change the trainee's knowledge from a two-dimensional to a 3-dimensional vision of the anatomy of the epidural region by using a standardized video recording. In this video clip, lumbar anatomy was explained by using vertebral models. During and after watching the video, trainees had to set up a plastic model of the epidural region with various plastic materials representing the ligaments. After the construction of the epidural model, more detailed information and comparison with the information contained in the anatomy texts and the micro- and macroscopic anatomy of the anatomical structure involved in the lumbar epidural block was provided. An instructor was present to assist the trainees in setting up the model and to answer questions.

#### 2.1.2. Module

The second module consisted of a video in order to familiarize the trainees with the materials and explain the basic principle of the epidural technique. After the video, the trainees performed a practical exercise to appreciate the increase and the loss of resistance with a Tuohy needle, a syringe, and a silicone cube. A discussion and clarification with the participants on the general principles ended this section.

#### 2.1.3. Module

The third module used an epidural simulator as a task trainer through which the Tuohy needle is passed with and without the help of the CompuFlo Epidural Trainer®. The aim was to let trainees appreciate and know how to recognize the differences in resistance offered by the fabrics encountered by having objective feedback. The CompuFlo Epidural Trainer®, based on Dynamic Pressure Sensing® technology, can detect pressure changes imperceptible by touch and presents visual and audible feedback allowing the trainee to accurately confirm the location of the needle and consistently discriminate between false and true loss of resistance encountered during the procedure, through the analysis of the graph and the acoustics of the instrument.

Using the CompuFlo Epidural Trainer®, the entry of the needle into the ligamentum flavum is indicated by a great increase in pressure on the visual display with a simultaneous increase in the pitch of the audible tone, while the entry of the needle into the epidural space results in a brisk drop in pressure and a distinct fall in the tone of the audio output. A drop in pressure sustained for more than 5 seconds is consistent with entry into the epidural space. Comprehensive information on the use of the CompuFlo Epidural® has been reported in previous studies to which we refer for more technical details [10, 11, 14, 15].

Typical curves, comparable with those obtained in humans [15] may be obtained, and graphs illustrating the procedure were recorded and eventually examined by each trainee performing the block, to discuss the correlation between the trainee's tactile sensations, the acoustic notification, and the visual display of the pressure waveforms. 

### 2.2. Learning Curve Assessment

After having received the initial training, all the trainees were admitted to the labor ward and started to practice labor epidural analgesia under the supervision of an instructor, blinded to the group assignment, in accordance with the Institutional practice.

A CUSUM learning curve was built for every trainee.

The CUSUM curve is a control chart to assess the outcomes of a series of consecutive procedures performed over the time. The graph obtained is plotted with the CUSUM value on the *y* axis and the number of consecutive attempts on the *x* axis.

A score of 0 was assigned to the CUSUM binary scale and the procedure was considered to be successful when both: (1) the epidural procedure was completed without any physical assistance from another staff member and (2) the epidural block provided effective analgesia, defined as a visual analogue pain scale (VAPS, 0 no pain; 100 worst pain) equal or less than 10 after 20 minutes from the administration of the epidural loading dose.

A score of 1 was assigned to the CUSUM binary scale and the procedure was considered to have failed when at least one of the following conditions was present: (1) the epidural procedure was completed with physical assistance from another staff member or when the instructor decided, after having interrupted the procedure performed by the trainee, to perform the procedure himself and (2) the epidural block provided ineffective analgesia.

The scores were assigned by the staff instructor, who was unaware of the group to which the trainee belonged.

### 2.3. Statistics

The performance improvement, defined as the completion of the procedure without the support of the tutor, was analysed through the observation of the learning curve CUSUM and the application of a parametric multivariate logistic regression model.

Cumulative failure charting and sequential probability ratio testing was performed. The number of cumulative failures was charted against the sequential attempt number for the series of patients. Control lines regarding acceptable or unacceptable performance were determined by first defining certain parameters. These parameters were as follows: the acceptable outcome rate (p0), the unacceptable outcome rate (p1), the Type I (false-positive) error rate (a), and the Type II (false-negative) error rate (b). Acceptable and unacceptable failure rates were set at 0.10 and 0.30, respectively. As standard for CUSUM analysis, Type I and Type II error rates were set at 0.10. Intermediate values (*a*, *b*, *P*, *Q* and *s*) were calculated as follows:

 
*a* = ln [(1−*β*)/*α*],
 
*b* = ln [(1−*α*)/*β*],
 
*P* = ln (*p*1/*p*0),
 
*Q* = ln [(1−*p*0)/(1−*p*1)],
 
*S* = *Q*/(*P* + *Q*).

The acceptable and unacceptable cumulative control lines were calculated, where *n* is the attempt number:

 
*h*0 = *s n*–*b*/(*P* + *Q*),
 
*h*1 = *s n*–*a*/(*P* + *Q*).

These control lines were also plotted on the chart. The crossing of either of these lines by the cumulative failure curve indicates acceptable or unacceptable performance in a series and hence conclusions can be drawn from the data.

Setting a fixed accepted and unaccepted failure rate, the achievement in performance (yes/no) and the number of attempts done to reach the improvement, represents the dependent variables under study whose correlations will be tested by applying logistic regression and *t*-test models.

### 2.4. Power Analysis

According to the primary end point, a priori power analysis (80% test power and 95% significance level) required a sample size of 42 subjects (21 for each group) to compare the mean numbers of attempts, applying a two-sample *t* test, and a total sample of 32 subjects to test a correlation between groups and performance achievement, applying a logistic regression model.

## 3. Results

All the enrolled participants completed the study. Considering an accepted and unaccepted failure rate of 10% and 30%, respectively, the number of subjects who achieved an improvement in performance (crossing the lower lines by the cumulative failure curve) were 8 trainees from the control group and 14 from the study group.

The probability of achieving an improvement was higher (*p* < 05) in the study group than in the control group, with an aOR of 3.25 (CI: 1.01; 12.1).

**Figure 1:**
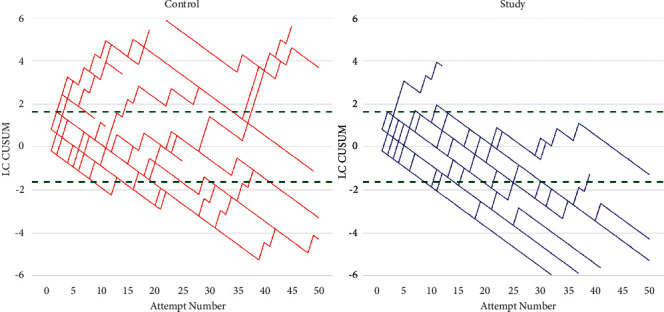
CUSUM curves of subjects in the control and study groups.

The average number of epidurals to reach the competence level performed by these subjects was 17 (±11) for the control group and 18 (±13) for the study group. There was no significant difference between the number of epidurals performed by the two groups before the expected performance was achieved. In Figure 1, the frequency distributions of the trainees who recorded an improvement in performance with CUSUM and the median number of epidurals made to achieve the improvement are reported.

In Table 1, the frequency distributions of the trainees who recorded an improvement in performance with CUSUM and the mean number (±SD) of epidurals made to achieve the improvement are reported. Logistic regression and *t* test were used to compare the groups. The descriptive statistics reported for different levels of accepted and unaccepted failure rate show a higher number of subjects who achieved the improvement in performance in the study group when compared to the control group and that the number of attempts required to record an improvement in performance with the CUSUM was not different between the groups, regardless of the established failure rate levels.

The proportion of subjects in the study group who completed the epidural without help was 1.21 (1.05–1.41) times the proportion of subjects who completed the epidural without help in the control group. The probability of completing the epidural block without any assistance was 21% higher in the study group than in the control group (*p* < 05).

## 4. Discussion

Using CUSUM analysis, our study found that anesthesia trainees demonstrated competence in epidural labor analgesia after a mean minimum case experience of 17 epidural blocks, based on a predefined acceptable failure rate of 10%. The new and significant finding of this study was that the percentage of trainees who reached the competence level was significantly greater in the group who underwent the structured epidural teaching model (67% vs 38%).

With regard to the number of attempts necessary to reach the proficiency level, our study reported different results as compared to those obtained in previous papers that have looked at competence in performing labor epidural using CUSUM analysis.

Kestin [16] studied novice (who had no previous experience in epidural blocks) and experienced trainees by collecting their data retrospectively. They used 5% and 10% as acceptable and unacceptable failure rates, defining failure as “failure to obtain analgesia or anesthesia for any reason”. The number of attempts made before crossing the boundary of the accepted failure rate ranged from 29 to 185 attempts. Only 4 out of 12 trainees (33%) achieved the competence level in an unspecified period of time.

Naik et al. [17] defined a successful epidural as “an independently placed epidural catheter that provided some degree of analgesia, without physical assistance from a staff anesthesiologist”. The trainees self-reported their score over a six-month training period. Using 10% and 15% as acceptable and unacceptable failure rates, they found that 10 out of 11 subjects (90%) achieved competency with the 57 median number of attempts during their six-month obstetric rotation.

Lew et al. [18] studied residents, with no prior experience, performing obstetric combined spinal-epidural. Their definition of success was a successful, unassisted procedure with a number of attempts equal or less than two. Competence was achieved by 19/24 residents (79%) after 40 combined spinal-epidural procedures in more than one year of training, based on a predefined acceptable failure rate of 20%. Data were collected retrospectively from the institutional database.

Differences between our study and previous ones are not surprising since each study used different acceptable and unacceptable failure rates and it is well known that establishing different acceptable failure thresholds in CUSUM analysis leads to a different minimum number of procedures needed to reach the competence level [19].

For this reason, we believe that the number of attempts *per se* is not a very sensible tool to evaluate performance since it is highly dependent on the setting of the CUSUM curve.

The definition of competence also varied between the studies as well as the duration of training. In addition, all previous studies evaluated the binary end point for the calculation of the CUSUM curve by using retrospective analysis of the institutional medical records or the self-assessment made by the trainees on their own, while our study was prospective, randomized, and blinded, in the sense that the observer who gave the scores was unaware of the group to which the observed trainee belonged.

Concerning our choice of the end points, we adopted clinical criteria that considered technical factors, such as the quality of analgesia (successful analgesia) and the incidence of additional intervention by the staff instructor, all important elements in evaluating the proficiency of a novice trainee. The quality of analgesia for the entire duration of labor was not considered a criterion of success because obstetric factors can affect the quality of analgesia and because our goal was to evaluate the competence in performing the block and not in managing the block during labor.

Setting different failure rates can produce different results, leading to confusion and inconsistency when comparing CUSUM results [19] and for this reason, after completing the study, we modified our threshold values as reported in Table 1. As expected, changing the acceptable and unacceptable failure rates determined the different mean number of procedures needed to reach the competence level.

However, the major result of our study—the greater percentage of trainees reaching the competence level after the structured epidural teaching model—was still observable and significant even if the criteria for acceptable and unacceptable failure rates were changed, and this makes our findings very robust.

Our study is not exempt from the same methodological limitations of any other CUSUM curve study [20]. However, in our case, the study was prospective and not retrospective, the binary scores were given by an independent, blinded observer and not self-assessed, the end points were clearly defined, and this may have compensated against the major criticisms made towards this statistical method.

One other limitation may also be that test performance depends on the difference between the adequate performance level and the acceptable deviation from that level and on the number of procedures during which the trainee is observed. The larger the difference and the longer the observation period, the better the test performance [21]. Our trainees made their institutional routine rotation in obstetric anesthesia for a six-month period and we cannot exclude that if they had had a longer training period, the percentage of trainees achieving the competence level, after the structured epidural teaching model would most likely have been even greater or more significant. Nevertheless, we have demonstrated the educational value of the SETM even in a limited period of time of training, and further studies with a longer period of training are encouraged.

Further multicenter studies involving several universities are ongoing in order to confirm the results of this study. Another future objective is whether the performance improvement associated with SETM, which is a formal structured and programed teaching, which also relies on new technologies such as the CompuFlo™ Epidural Trainer instrument, remains stable over time.

In conclusion, we have demonstrated that the use of the structured epidural teaching model (SETM) may improve the lumber epidural block learning curve (CUSUM) in novice, entirely inexperienced, anesthesia trainees.

## Figures and Tables

**Table 1:** Effects of changing the levels of accepted and unaccepted failure rate on the frequency distributions of the trainees who recorded an improvement in performance with CUSUM and on the mean number (±SD) of epidurals carried out to achieve the improvement.

Failure rate	*Number of trainees*					*Number of epidurals*		
Accepted	Not accepted	Study (21)	Control (21)	OR (CI 95%)	*p*.value	Study	Control	*p*.value
10	30	14 (67%)	8 (38%)	3.25 (1.02; 12.1)	<.05	18 (±13)	17 (±11)	>.05
10	40	16 (76%)	16 (76%)	1 (0.23; 4.26)	>.05	12 (±8)	12 (±9)	>.05
20	30	9 (42%)	7 (33%)	1.5 (0.43; 5.4)	>.05	28 (±10)	31 (±10)	>.05
20	35	13 (61%)	7 (33%)	3.15 (1.01; 11.3)	<.05	17 (±7)	19 (±10)	>.05
20	40	16 (76%)	14 (67%)	1.6 (0.42; 6.51)	>.05	13 (±6)	18 (±13)	>.05

## Data Availability

The data used to support the findings of this study are available from the corresponding author upon request.
